# Novel Mitochondrial Cytopathy Causing Mitochondrial Encephalomyopathy With Lactic Acidosis and Stroke-Like Episodes Syndrome and Tubulointerstitial Nephropathy

**DOI:** 10.7759/cureus.66722

**Published:** 2024-08-12

**Authors:** Saurav Dhawan, Abdel H Musa, Khyathi Mantripragada

**Affiliations:** 1 Internal Medicine, Manchester University National Health Service (NHS) Foundation Trust, Manchester, GBR; 2 Internal Medicine, Stockport National Health Service (NHS) Trust, Stockport, GBR

**Keywords:** novel mutation, mitochondrial disorder, stroke, kidney transplant, melas

## Abstract

Mitochondrial cytopathies, predominantly MT-TL1 mutations and, to a lesser extent, MT-ND5, have been associated with mitochondrial encephalomyopathy with lactic acidosis and stroke-like episodes (MELAS), manifesting as multi-organ dysfunction. This is just the second instance of MELAS secondary to the pathogenic novel m.13091T>C variant of MT-ND5. Moreover, nephropathy associated with MT-ND5 mutation has only been reported in nine cases so far.

A middle-aged man presented in a state of acute confusion with speech difficulty with both receptive and expressive aphasia. He had a background of refractory seizures, chronic atypical migraine, childhood-onset optic neuropathy, and end-stage renal disease requiring renal transplant. During admission, he had episodes of aggression and paranoid beliefs.

Magnetic resonance (MR) imaging of the head showed multiple areas of cortical abnormality, unusual for age, including a large frontal infarct crossing arterial boundaries. Cerebrospinal fluid (CSF) protein and lactate were high, whereas, the electroencephalography (EEG) result was normal. Muscle biopsy mitochondrial DNA gene sequencing derived novel MT-ND5 gene variant m.13091T>C p.(Met252Thr). Kidney biopsy previously had shown interstitial fibrosis and tubular atrophy. He was managed as acute ischaemic stroke along with a combination of clobazam, levetiracetam, and eslicarbazepine for seizures.

MELAS typically presents with seizures, stroke-like episodes, cortical visual loss, and recurrent migraine headaches. The previous reported case of m.13091T>C mutation followed a similar progression, however, there was no associated nephropathy and normal visual acuity. Kidney transplants in affected patients of MELAS have been associated with a high survival rate. MT-ND5 mutation-associated nephropathy has shown a variable manifestation, either as focal segmental glomerular sclerosis (FSGS) or tubulo-interstitial disease.

## Introduction

Mitochondrial cytopathies are increasingly being recognized globally and can range in presentation from single-organ to multi-organ dysfunction. MELAS (mitochondrial encephalomyopathy with lactic acidosis and stroke-like episodes) is one of the most common mitochondrial syndromes presenting commonly with neurological manifestations and rarely involving kidneys [1]. We describe a case of MELAS-like syndrome that presented with stroke-like features and seizures and had a background of kidney transplantation in his 40s and childhood-onset optic neuropathy. Investigations revealed old and new infarcts on magnetic resonance imaging (MRI) of the head, cerebrospinal fluid (CSF) analysis consistent with MELAS, prior renal biopsy showing end-stage nephropathy and muscle biopsy demonstrating the novel variant while electroencephalogram (EEG) was essentially non-specific. He was treated for acute infarct and seizures with different anti-epileptic drugs. Pathogenic variants of the MT-TL1 gene have been largely implicated with disease manifestation. MT-ND5 mutation has previously been known to be associated with MELAS, LHON (Leber's hereditary optic neuropathy), and Leigh disease [2]. The patient was found to be positive for a novel mutation in the mitochondrial MT-ND5 gene. MT-ND5 mutation manifesting as MELAS in association with nephropathy is much less recognized with only nine such cases reported so far. MELAS-like features with tubulointerstitial renal disease due to the novel pathogenic variant m.13091T>C of this gene is a rare occurrence, this being only the second such case to be reported, making it a very rare case among this group of diseases. A high index of suspicion is required while diagnosing these patients with atypical multi-organ dysfunction.

## Case presentation

A man in his 40s presented to the emergency department in a state of acute confusion with speech difficulty. He was found wandering inappropriately in the streets around his residence. He was haemodynamically stable with the following observations at presentation - blood pressure 132/85, pulse rate 70/minute, respiratory rate 17/minute, oxygen saturation (SpO2) 99% in room air. On examination, he didn’t seem confused but was aphasic with both receptive and expressive components. The rest of the neurological and systemic examination was unremarkable, including the site of the right-sided transplanted kidney. He had a background of chronic kidney disease since his 30s that had progressed to end-stage renal disease in the next decade. He had undergone a renal transplant (first donation after circulatory death kidney transplant) recently. He also had a history of recurrent vascular problems, namely, controlled hypertension and mitochondrial cytopathy, causing a stroke that had been thrombolysed a few years back. Additionally, he had a history of focal motor seizures affecting his left foot (treated with levetiracetam, lacosamide, and clobazam), chronic atypical migraine (refractory to three different preventive medications, while benefitting from botulinum toxin), and childhood-onset optic neuropathy. During admission, he had episodes of aggressive behaviour and expressed paranoid beliefs about the staff. He had multiple migraine episodes with aura during his stay. His speech improved significantly during the course of his admission with improved coherence and grammar; however, he continued to have paranoid delusions that also started to resolve eventually after a series of medication alterations. His thought content was normalised with no evidence of delusions and good recall.

He had previously documented allergies to gabapentin, statins, pregabalin, and amitriptyline. His father had rheumatoid arthritis and his mother had scleroderma. His sister had some degree of hearing loss since childhood. His brother had asthma. His brother’s son was diagnosed with spinal muscular atrophy type 2 (SMA). He denied being aware of any other similar disorders in the family.

Investigations

His blood investigations at the time of admission were unremarkable with the renal function at baseline levels (Table 1). CT Head demonstrated interval right frontoparietal, left temporoparietal, occipital, cortical, and subcortical hypodense lesions, likely representing established non-haemorrhagic infarcts.

**Table 1:** Relevant blood investigations at the time of presentation eGFR: Estimated glomerular filtration rate, CRP: C-reactive protein, ALT: Alanine aminotransferase, ALP: Alkaline phosphatase, Anti MPO: Anti-myeloperoxidase antibodies, Anti PR3: Anti proteinase 3, Ig: Immunoglobulin, Anti TTG: Anti-tissue transglutaminase antibodies.

	Value	Normal reference range	Units
WBC	7.0	4.0 - 11.0	X 10^9^ / L
Haemoglobin	128	130 - 180	g/ L
Neutrophils	4.21	1.80 - 7.50	X 10^9^ / L
Lymphocytes	1.89	1.00 - 4.00	X 10^9^ / L
Sodium	134	133 - 146	mmol/ L
Potassium	4.6	3.5 - 5.3	mmol/ L
Urea	6.5	2.5 - 7.8	mmol/ L
Creatinine	113	59 - 104	micromol/ L
eGFR	68	>90	mL/min/1.73m^2^
CRP	9	0 - 5	mg/ L
ALT	16	1 - 50	IU/ L
ALP	36	30 - 130	U/ L
Bilirubin	8	0 - 21	micromol/ L
Calcium	2.55	2.20 - 2.60	mmol/ L
Phosphate	1.47	0.80 - 1.50	mmol/ L
Magnesium	0.68	0.70 - 1.00	mmol/ L
Anti-nuclear Antibody	Negative		
Anti-centromere	<0.2	0.0 - 0.9	AI
Anti MPO	<0.2	0.0 - 0.9	AI
Anti PR3	<0.2	0.0 - 0.9	AI
Complement C3	1.75	0.75 - 1.65	g/ L
Complement C4	0.38	0.14 - 0.54	g/ L
Electrophoresis	No paraprotein detected		
IgG	8.72	6.00 - 16.0	g/ L
IgA	2.06	0.80 - 2.80	g/ L
IgM	0.60	0.50 - 1.90	g/ L
Rheumatoid Factor	<10	0 - 13	kIU/ L
Anti TTG	<0.5	0.0 – 14.9	kU/ L

**Figure 1:**
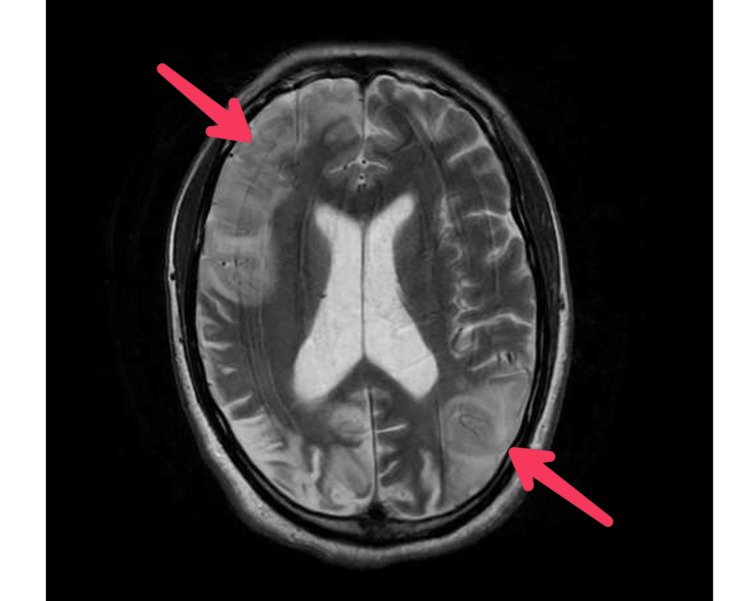
Acute infarcts as seen on initial MRI of the head

MRI Head showed multiple areas of cortical abnormality, unusual for age, old as well as new, including a large frontal abnormality crossing arterial boundaries (Figure 1). CSF analysis revealed high CSF protein (0.67 g/L) and lactate (3.4 mmol/L) with normal CSF glucose (3.7 mmol/L). Homocysteine levels were slightly elevated (17 mmol/L). CSF microscopy and culture showed no growth. CSF screen also returned negative for Herpes simplex virus (HSV1, HSV2), Varicella Zoster virus, Enterovirus, Parechovirus, Meningococcus, Meningococcus B and Pneumococcus (Table 2). Alpha-galactosidase was negative. EEG didn’t show any evidence of prominent encephalopathy, subclinical seizures, or epileptiform discharges.

**Table 2:** Relevant CSF investigations CSF: Cerebrospinal fluid; HSV: Herpes Simplex Virus; PCR: Polymerase Chain Reaction; VZV: Varicella Zoster Virus

	Value	Normal reference range	Units
CSF Appearance	Clear, colourless		
CSF Glucose	3.7	2.2 – 4.4	mmol/ L
CSF Protein	0.67	0.15 – 0.45	g/ L
CSF Lactate	3.4	1.1 – 2.4	mmol/ L
CSF WBC count	<3		X 10^6^ / L
CSF RBC count	<3		X 10^6^ / L
CSF Microscopy	No organisms seen		
Meningococcal PCR	Negative		
Pneumococcal PCR	Negative		
HSV1 PCR	Negative		
HSV2 PCR	Negative		
VZV DNA PCR	Negative		
Enterovirus RNA PCR	Negative		
Parechovirus RNA PCR	Negative		

**Figure 2:**
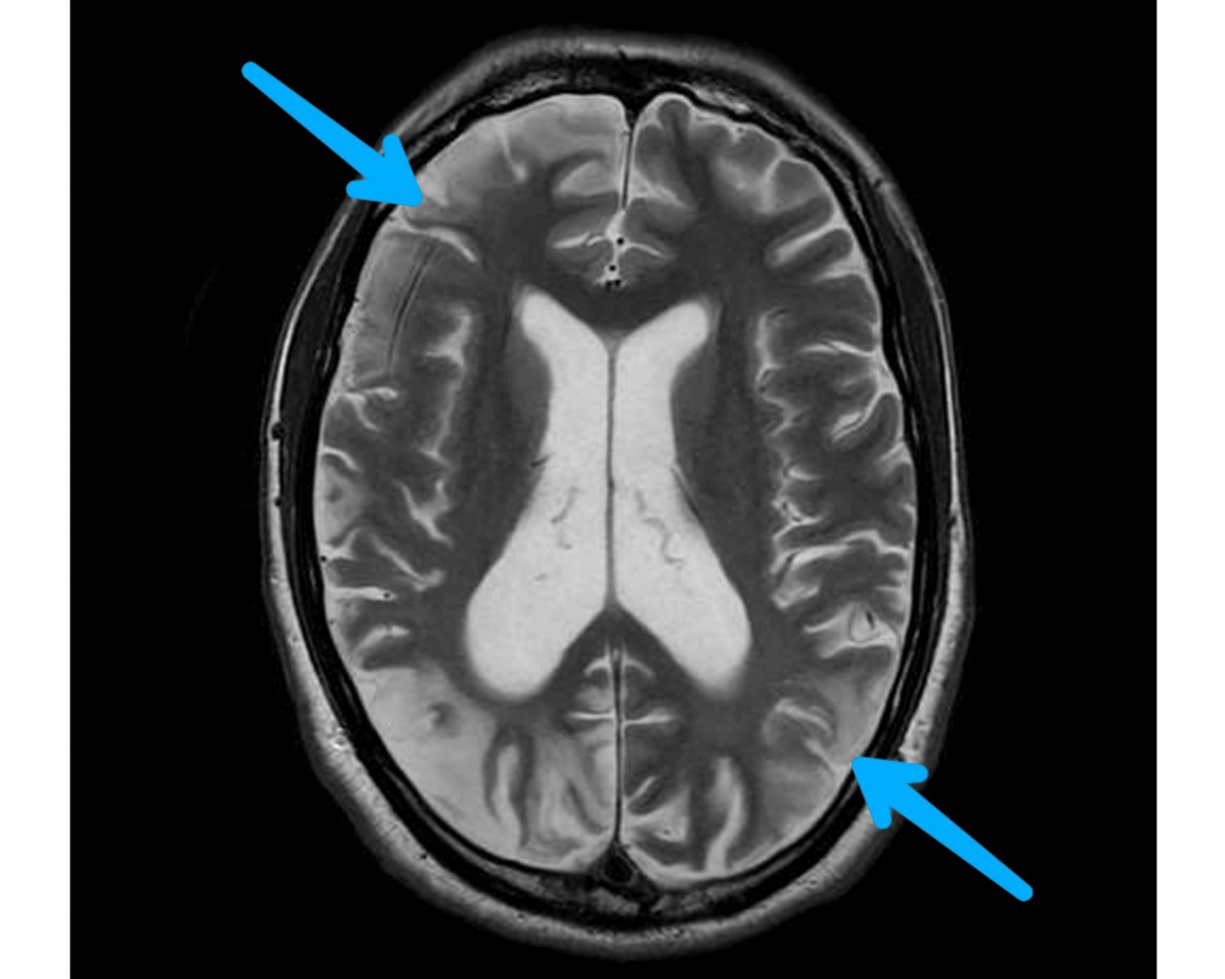
MRI head image after three weeks Interval resolution of infarcts seen in repeat MRI head after three weeks.

A repeat MRI head was requested at an interval of four weeks that showed significant interval resolution of previously noted infarcts, although some persistent signal abnormality was seen in the left parietal region (Figure 2). A repeat EEG was requested that didn’t show any interictal epileptiform activity and no significant change from the previous recording. On his routine blood examination, hyponatraemia of 121mmol/L was found and considered likely due to the syndrome of inappropriate antidiuretic hormone secretion (SIADH) secondary to medications. Hyponatraemia developed after starting treatment with Eslicarbazepine with a urine osmolality of 486 mosm/kg and urine sodium of 45 mmol/L with a normal extracellular fluid volume status. Sodium levels improved with fluid restriction and dosage adjustments to ongoing medications.

A young stroke screen conducted seven to eight years ago was negative. A blood mitochondrial DNA analysis had returned negative for MELAS, myoclonic epilepsy with ragged red fibers (MERRF), NARP (neuropathy, ataxia, and retinitis pigmentosa), and LHON. Muscle biopsy derived mitochondrial DNA gene sequencing novel MT-ND5 gene variant m.13091T>C p.(Met252Thr), described as mitochondrial mutation of unknown significance. There was an intermediate level of heteroplasmy in muscle (54%) and a low level (10%) in his blood-derived DNA. A kidney biopsy around 10 years ago had shown non-specific findings with interstitial fibrosis and tubular atrophy, considered to be secondary to small vessel ischaemia.

Differential diagnosis

He was admitted with a provisional diagnosis of a new Broca’s area infarct. Other differentials included encephalitis (since he was on immunosuppression for his renal transplant), postictal confusion (considering the possibility of having had seizures), posterior reversible encephalopathy syndrome (PRES), and medication induced confusion, however, all of these were considered unlikely. Broca’s infarct was unlikely in the context of both expressive and receptive aphasia. He didn’t have any signs suggestive of encephalitis or meningitis with a negative infection screen on blood and CSF investigations as detailed above. Electrolytes were within normal range at presentation thus excluding dyselectrolytaemia as a factor contributing to encephalopathy. He developed hyponatraemia during recovery after starting eslicarbazepine, but it didn’t contribute to or worsen existing symptomatology. He had been on immunosuppressive medications, namely, tacrolimus and mycophenolate mofetil which is a risk factor for PRES. However, he didn’t have uncontrolled hypertension at the time of presentation or during the hospital stay. He had not been on anti-hypertensive medications. His renal transplant was functioning well with no signs of decompensation. Moreover, there were no vasogenic oedema or hyperintense signals on the T2 MRI sequence in parieto-occipital or other lobes, which are findings expected in a patient of PRES. There was also evidence of definite cortical damage from previous insults. MRI head suggested multiple new unusual infarcts. The findings of CSF analysis were in keeping with those found in mitochondrial cytopathy. Stroke in non-arterial distribution in the context of mitochondrial cytopathy emerged as the likeliest diagnosis. The patient’s behavioural disturbance, psychosis, and visual hallucinations were considered to be secondary to possible non-convulsive subclinical seizures. However, uncertainty remained as the genetic workup returned negative results for all pathogenic variants of MELAS with the genetic analysis report classifying the detected variant as being of unknown significance. Moreover, MELAS had typically been associated with focal segmental glomerular sclerosis (FSGS) in patients with renal involvement with few tubulointerstitial diseases known. This patient had a fairly early onset tubulointerstitial end-stage renal disease not attributable to any other cause. Thus, it was concluded that the patient’s mutation was indeed pathogenic. Hence, the diagnosis of MT-ND5-associated mitochondrial cytopathy due to the pathogenic variant m.13091T>C causing MELAS-like syndrome with tubulointerstitial nephropathy and childhood-onset optic neuropathy was confirmed. 

Treatment

He was started on aspirin 300 mg followed by clopidogrel for acute ischaemic stroke. It was debated whether his immunosuppressive medications need to be held, however, they were continued since there was no other clinical or laboratory evidence of encephalitis. He was started on clobazam 10 mg twice daily for subclinical seizures in addition to levetiracetam 1 gram twice daily that he had been previously taking. A low threshold was kept for the escalation of anti-epileptics. Intravenous L-arginine was not used due to the poor evidence for its use in such patients. Clobazam was later increased to 20 mg twice daily. Subsequently, eslicarbazepine was started at 400 mg daily and increased to 800 mg daily after three days. We had to alter his medications due to hyponatraemia. Levetiracetam was reduced to 750 mg twice daily. Lacosamide was not preferred owing to its cardiac side effects. Levetiracetam was further reduced to 500 mg twice daily, and later, eslicarbazepine was reduced to 400 mg twice daily. Tacrolimus levels were regularly checked and titrated. Renal graft function remained stable throughout admission. He was discharged on these doses with outpatient Neurology, Neuropsychiatry, and renal transplant follow-up.

Outcome and follow-up

MRI head findings were discussed at a neuroradiology multidisciplinary team meeting and it was concluded that the suggested findings were in keeping with MELAS-like syndrome while also correlating them with his known genetic mutation and clinical presentation. The patient had risk factors for PRES (on tacrolimus, mycophenolate mofetil). However, the pattern of radiological changes on the MRI head didn’t favour it. It was not possible to arrange for testing of his mother or other family members to help classification of the genetic change. He was due for follow up at neurology outpatient clinic, however, represented to hospital with increased seizure frequency - focal seizures that progress to generalised seizures.

## Discussion

Primary mitochondrial disorders are genetic disorders arising secondary to different pathogenic variants in genes that code for the mitochondrial respiratory chain. MELAS (mitochondrial encephalomyopathy, lactic acidosis, and stroke-like episodes) is commonly seen in individuals between 2-40 years of age [1]. The most common mutation associated with MELAS is related to pathogenic variants of the MT-TL1 gene (>80%) whereas MT-ND5 contributes to only less than a tenth of these cases. It has been noted that the occurrence of heteroplasmy in mtDNA disorders can cause variable spread of mutated mtDNA [2]. More than 90% of patients exhibit symptoms like seizures (focal and generalised) and stroke-like episodes (like partially reversible aphasia and motor weakness), while a majority also experience cortical visual loss and recurrent migraine headaches. In order to diagnose MELAS, a criterion has been established based on the presence of symptoms in two categories as detailed below (Table 3) [3].

**Table 3:** Diagnostic criteria for MELAS

Category A (>=2)	Category B (>=2)
Headaches associated with vomiting	1. Raised CSF/plasma lactate
Seizures	2. Muscle biopsy demonstrating mitochondrial abnormalities
Hemiplegia	3. Presence of a pathogenic variant related to MELAS
Cortical vision loss	
Neuroimaging evidence of acute focal lesions	

Renal involvement is uncommon in MELAS patients, mostly presenting as either tubulopathy, interstitial nephritis, FSGS (most common), or cystic pathology [4-7]. It is most linked to MT-TL1 mutation (m.3243A>G) [8]. Kidney transplant in these patients has been associated with a 100% survival rate with a follow-up range extending up to 12 years [9]. However, MT-ND5 mutation-associated adult onset nephropathy is extremely rare and is described in only nine cases so far with variable manifestations consisting of a mix of FSGS and tubulointerstitial disease (Table 4) [10-14].

**Table 4:** Renal nephropathy distribution in MT-ND5 mutation till date

FSGS	Tubulo-Interstitial
40%	60%

The previously reported case of m.13091T>C mutation followed a similar progression, presenting initially with chronic migraines followed by recurrent stroke-like episodes and secondary epilepsy. However, there was no associated nephropathy and normal visual acuity, although ophthalmological tests showed bilateral optic disc pallor and reduced retinal nerve fibre layer thickness across all four quadrants [15].

## Conclusions

Globally, mitochondrial syndromes are now being increasingly diagnosed, causing varied levels of presentation from single-organ to multi-organ dysfunction. Many known genetic mutations, especially in the MT-TL1 gene, are responsible for such presentations. MT-ND5 mutation is a rare but known cause of MELAS. Disease manifestation can also occur secondary to certain novel variants like m.13091T>C, as in this case, which may be reported as being of unknown significance. Moreover, MT-ND5 mutations can rarely cause adult-onset nephropathy, either isolated or associated with MELAS and presenting either as FSGS or tubulo-interstitial disease. Therefore, it is important to consider mitochondrial cytopathies as a differential in patients less than 40 years of age presenting with stroke-like episodes, seizures, recurrent migraine headaches, and nephropathy.
